# Giant cell arteritis (temporal arteritis): A report of four cases from north east India

**DOI:** 10.4103/0972-2327.42940

**Published:** 2008

**Authors:** Judy Laldinpuii, Pratap Sanchetee, Adityendra Lal Borah, Manash Ghose, Nomal Chandra Borah

**Affiliations:** Department of Neurology, Institute of Neurological Sciences, GNRC Hospitals, Dispur, Guwahati, India; 1 Department of Neuropathology, Institute of Neurological Sciences, GNRC Hospitals, Dispur, Guwahati, India

**Keywords:** Giant cell arteritis, polymyalgia rheumatica

## Abstract

Giant cell arteritis (GCA) is a common disease of the geriatric age group in the western world, with a prevalence of 0.2% in the fifty plus age group. It is an important cause of morbidity, with irreversible visual loss being the most ominous complication. This diagnosis is an important consideration in all cases of new onset headache in elderly subjects. Reports of giant cell arteritis are few and far between in the Indian subcontinent. In this report, we describe the clinical details of four cases of giant cell arteritis, detected at Guwahati, Assam. The four patients were in the 70-82 age group. Sex distribution was equal. All of them had polymyalgia rheumatica (PMR), with one case displaying an initial presentation as only PMR. Cardinal manifestation was a severe headache, frequently accompanied by scalp allodynia and abnormalities of the superficial temporal artery (STA) on examination. STA biopsy yielded histopathological confirmation in three patients. Permanent visual loss was noted in one patient. These cases highlight the importance of assessing the possibility of giant cell arteritis through appropriate clinical history, estimation of acute phase reactants and the judicious use of superficial temporal artery biopsy, to clinch the diagnosis.

## Introduction

Giant cell arteritis (GCA) is a chronic inflammatory vasculitis involving the medium and large sized vessels.[1] Prevalence of the disease is 0.2% in the fifty plus age group.[2] This disease predominantly affects the elderly, with peak incidence occurring in the 70-80 age group. It is an important cause of secondary headaches as well as a preventable cause of blindness in the elderly.

The diagnostic criteria for giant cell arteritis, as laid down by the American College of Rheumatology, demands the fulfillment of at least two of the following five criteria (i) age more than 50 years (ii) new headache (iii) superficial temporal artery (STA) tenderness or decreased pulsation (iv) elevated ESR more than 50 mm in the first hour, and (v) abnormal findings on temporal artery biopsy.[3] To the best of our knowledge, cases of giant cell arteritis has been reported from the Indian subcontinent.[4–7] We would like to report our experience with four cases of GCA, who were diagnosed and treated at our institute in the last two years [Table 1].

**Table 1:** Summary of clinical findings in 4 cases of giant cell arteritis

Characteristic	Case 1	Case 2	Case 3	Case 4
Age	70	75	82	70
Sex	Male	Male	Female	female
Ethnicity	Assamese	Tripuri	Khasi	Nepali
Duration of symptoms (months)	6	1	6	12
Headache intensity	Moderate	Severe	Severe	moderate
PMR	Present	Present	Present	present
Constitutional symptoms	+	+	++	+
Visual loss	No	Yes AION one eye	No	no
Jaw claudication	No	Yes	Yes	no
Temporal artery tenderness	Present (moderate)	Present (severe)	Present (severe)	Present (moderate)
ESR/CRP	50/+	35/+	185/+	135/+
Hb (gm%)	9.8	10	8	9.6
Rheumatoid factor	Negative	Negative	Negative	Negative
ANA	Negative	Negative	Negative	Negative
STA biopsy	Arteritis	Arteritis	Atherosclerotic	Arteritis

## Case Reports

### Case 1

A 70-year-old male, previously in good health, presented with a two-month history of pain in the shoulder joints, upper back and chest, which was aggravated by motion. He had early morning stiffness with constitutional symptoms. Laboratory tests were as tabulated. A possibility of polymyalgia rheumatic (PMR) was considered and response to low dose steroid was gratifying. He discontinued drugs after one month and remained well for the next five months. At that stage, he had recurrence with additional complaint of left-sided headache. The left STA was tender and thickened. A possibility of PMR with GCA was considered. A left STA biopsy was performed, which confirmed the diagnosis. Steroids were administered at an oral dose of 50 mg per day and tapered gradually, with excellent clinical response over a follow up period of one year.

### Case 2

**Figure 1:**
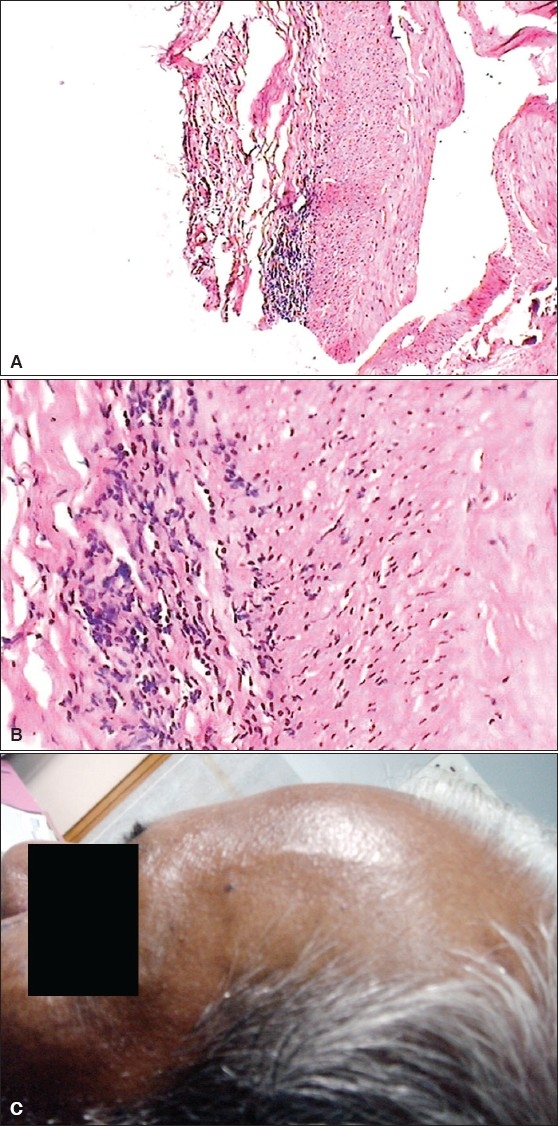
(A) H and E stained low power sections of the STA of Case 2 showing infiltration with chronic inflammatory cells in the arterial adventitia and the media. Note is made of arterial wall calcification. (B) Hand E stain close up high power view of STA of the same patient showing a focus of intense inflammatory activity with chronic mononuclear cells mainly in the arterial media and in the adventitia. (C) Prominent superficial temporal artery of case 1. This artery was tender to touch

A 75-year-old male presented with headache of six weeks' duration. Pain was predominantly over the right hemicranium, with the maximum being over the right temple. Pain was excruciating in intensity. There was severe allodynia over the area. There was jaw claudication. Constitutional symptoms were present. About two weeks after the onset of headache, he developed blurred vision in the right eye; an ophthalmological examination revealed anterior ischemic optic neuropathy (AION). Examination and salient laboratory findings are as tabulated in Table 1. Diagnosis of GCA was confirmed with STA biopsy [Figure 1]. He was treated with intravenous methyl prednisolone, in a dose of 1 gm/day for three days, followed by oral steroid in tapering doses. His headache and constitutional symptoms improved rapidly. However, visual acuity remained unchanged. He had recurrence of mild headache, three months after he stopped the steroids by himself, and improved with re- institution of steroid.

### Case 3

An 83-year-old lady, complained of insidious onset generalized weakness of six months' duration, with multifocal ill-defined body pains, loss of appetite, loss of weight and prostration to a bed bound state. Two months prior to presentation, she had additional complaints of severe, distressing headache, which was maximal over the left temple. She avoided solids due to jaw claudication. She was admitted to a nursing home, where the physician started Anti tubercular therapy (ATT), on suspicion of tuberculosis. The clinical and salient laboratory findings are tabulated. The STA biopsy failed to demonstrate inflammation, and the patient refused a repeat biopsy. Therapeutic trial with oral steroids at 1 mg/kg was instituted, which caused complete relief of headache and jaw claudication and partial improvement in the systemic symptoms. At six months, she was reported by attendants to be free of the headache, but house-bound due to weakness.

### Case 4

A 70-year-old lady, with a well controlled hypertension, presented with a history of headache, arthralgias and malaise of one year duration. Headache was global, with maximum intensity over the left temple. She had been on various migraine prophylaxes and analgesics, without relief. She had associated pain and stiffness in multiple large and small joints, with marked early morning stiffness. She was depressed and slept poorly. A diagnosis of PMR with GCA was made. Symptoms improved within 72 hours of starting oral steroid at 1 mg/kg/day. She had a relapse of the same symptoms, after a year of stopping the therapy on her own. Re-treatment was successful

## Discussion

The first clinical description of GCA was made by Hutchison in 1890.[8] The typical pathology in the temporal arteries was described by Horton *et al.* in 1930.[1] The commonest symptom of GCA is headache (72%), which is maximal over the temple and the occipital regions. Scalp tenderness may be present. The STA, occipital, post auricular or facial arteries may demonstrate thickening, nodularity, tenderness or erythema. The involvement of larger vessels, particularly the subclavian and axillary vessels, as well as a seventeen-fold increase in the risk of the development of thoracic aorta aneurysm is reported. Jaw claudication is seen in 40% of the cases of GCA. Jaw claudication was present in two of our patients (Cases 2 and 3).

PMR is characterized by proximal and axial joint arthralgias, early morning stiffness, aggravation of pain by movement, elevated ESR and response to low dose steroid.[9] It is more common than GCA, with reported prevalence up to one in 133, in the population at risk (above 50 years).[10] There is an interesting yet incompletely understood relationship between these two conditions. Studies have shown that 16–21% of the patients with PMR develop GCA.[10] Conversely, PMR is noted in 58% of cases diagnosed with GCA.[11] GCA presenting as apparently isolated PMR, without cranial symptoms or only minimal cranial symptoms, also carries a 27.4% increased risk of ischemic episodes, as per available retrospective data.[12]

The patients we have described are all over seventy years of age and had presented with typical excruciating headaches, well localized to the temples. A tender STA, PMR and response to steroids were features suggesting the diagnosis in all four patients.

Visual loss is the most dreaded complication of GCA, seen in 20% of the cases.[13] Case 2 developed AION of the left eye, within one month of the onset of symptoms. Visual loss is reported to occur due to ischemia in the optic nerve and tracts, mostly because of vasculitis of the ophthalmic and posterior ciliary arteries. The neurological manifestations in GCA include neuropathies (14%), which may be polyneuropathy or mononeuropathies, transient ischemic attacks or stroke (7%) and neuro rheumatical symptoms like hearing loss and vertigo (7%). Uncommon manifestations include scotomas (5%), tongue claudication (4%), depression (3%), diplopia (2%) and tongue numbness (2%).[14] Laboratory findings include elevated ESR and C reactive protein. However, ESR may be normal in 22.5% of biopsy proven GCA cases.[15] A similar situation was seen in Case 2, where there was normal ESR but confirmed GCA.

Harvesting a generous segment of STA (4–6 cm) and subjecting the material to meticulous examination of the artery with serial sub sectioning remains the gold standard of diagnosis. In negative cases, sampling the other STA to repeat biopsy may yield a positive response. Taking all these precautions, the incidence of false negative biopsy may be reduced to less than 10%.[16] The histopathological changes in the temporal arteries include luminal stenosis, intimal proliferation and disruption of internal elastic lamina by mononuclear cell infiltrate. The classic histopathological picture of giant cells located at the junction between intima and media is seen in 50% of the cases. The remaining show a panarteritis with mixed inflammatory cell infiltrate. The involvement is typically patchy (skip lesions). In the three patients with positive STA biopsy, we established a panarteritis picture. None of the STA biopsies studied revealed giant cells. Though we took pains to ensure adequate length of STA biopsy in the patients, repeat biopsy consent could not be obtained in Case 3, who was biopsy negative. In view of her typical presentation, we concluded that the negative biopsy may be due to skip lesions. Patients of suspected GCA, who have typical symptoms of GCA but are biopsy negative, are described to have less constitutional symptoms, less arterial wall abnormality and have lower chances of ischemic complication, as compared to biopsy positive cases.

Duplex sonography of the temporal arteries, with demonstration of a characteristic dark halo around the artery, is emerging as a viable alternative or a complement to the gold standard in non-invasive diagnosis of the condition.[17] It has a reported sensitivity of 73% and specificity of 100%. The demonstration of bilateral dark halos correlates well with development of ischemic complications in GCA and also has good histopathological correlation with biopsy results. [(18) F] Flourodeoxyglucose (FDG) PET, a non invasive, metabolic imaging modality based on the regional distribution of [(18) F] Flourodeoxyglucose (FDG) PET has a promising role in demonstrating large vessel arteritis, especially in active disease.[18] This modality is superior to morphological imaging modalities such as MRI in picking up more affected areas and also in discriminating vasculitis from atherosclerosis. High resolution contrast MRI studies can demonstrate vessel inflammation with good results (sensitivity 80.6% and specificity 97%).[19] These modalities correspond well to symptoms, acute phase reactant levels and with histopathology; they may supplement/obviate the need for vessel biopsy.

GCA typically responds rapidly and completely to steroids in doses of 40-60 mg per day. The steroid response was immediate and satisfactory in all the cases described here, though visual loss in Case 2 did not respond, which is the usual scenario. Steroids need to be started as soon as the diagnosis is suspected. There is no justification for withholding treatment pending biopsy, as histopathology does not show significant change within the first two weeks of steroid institution.[20] Typically, headache, fatigue and PMR symptoms respond within days, with gradual normalization of ESR and C Reactive protein (CRP) by 2–4 weeks. After symptomatic improvement, steroids are continued in tapering doses, guided by symptoms and ESR for maintenance levels. The duration of therapy is typically prolonged up to two years; patients with relapsing course may need longer course of treatment. Considering the side effects of steroid in this fragile age group, steroid sparing agents like Methotrexate and Azathioprine have also been used in chronic cases or in patients suffering from side effects. Intravenous methyl prednisolone is reserved for patients with recent or impending visual loss.[9]

In retrospective studies, low dose aspirin as well as anti-coagulation have been shown to have a positive role in decreasing ischemic complications of GCA, mainly visual loss and strokes, without any increase in the risk of hemorrhage.[21,22]

Unusual presentations of GCA that have been reported from the Indian sub continent earlier include a report of GCA, possibly triggered by psoralen therapy,[4] GCA as a cause of jaw claudication,[5] as an unusual cause of pyrexia of unknown origin in the elderly[6] and finally, as a cause of AION.[7]

The four cases highlighted in our report (with the exception of Case 2 who had a fairly abrupt course) had been symptomatic many months and had multiple contacts with physicians. However, the actual diagnosis was not considered and alternate diagnosis like migraine and tuberculosis were suspected and management instituted along those lines. This suggests that GCA is not a frequently considered cause of new onset headache in the elderly, at the level of primary physicians and ophthalmologists. New onset headache in an elderly patient should prompt a detailed assessment for the possibility of GCA (amongst other possibilities). PMR symptoms and STA abnormalities, especially with elevated ESR, are definite red flags. The gold standard diagnostic tool - STA biopsy - is a safe out-patient procedure and should be employed wherever the clinical possibility exists. Meticulous histopathological sampling and study are mandatory. A positive biopsy gives the physician the confidence to proceed with the aggressive steroid therapy that is required in this condition. Emerging modalities for demonstration of vasculitis include duplex ultrasound, high resolution MRI and FDG Pet studies. They have the advantage of being non-invasive and are also invaluable in biopsy negative/cryptic cases of GCA.
